# Nomenclatorial changes and redescriptions of three of Navás’ *Leucochrysa (Nodita)* species (Neuroptera, Chrysopidae)

**DOI:** 10.3897/zookeys.92.828

**Published:** 2011-04-28

**Authors:** Tauber Catherine A., Albuquerque Gilberto S., Tauber Maurice J.

**Affiliations:** 1 Department of Entomology, Comstock Hall, Cornell University, Ithaca, NY 14853–2601 and Department of Entomology, University of California, Davis, CA 95616; 2 Laboratório de Entomologia e Fitopatologia, CCTA, Universidade Estadual do Norte Fluminense, Campos dos Goytacazes, Rio de Janeiro, Brazil 28013–602

**Keywords:** Leucochrysini, neotropical lacewings, taxonomy

## Abstract

Three species that Navás described – *Leucochrysa (Nodita) azevedoi* Navás, 1913, *Leucochrysa (Nodita) camposi* (Navás, 1933) and *Leucochrysa (Nodita) morenoi* (Navás, 1934) – have received recent taxonomic attention. All three have many similar external features; indeed Navás himself, as well as subsequent authors, have confused the species with each other. Here, (a) misidentifications are corrected; (b) a neotype of *Leucochrysa azevedoi* is designated; (c) *Leucochrysa (Nodita) morenoi*, previously synonymized with *Leucochrysa (Nodita) camposi*, is recognized as a valid species [**Reinstated status**] All three species are redescribed and illustrated, with special emphasis on the types. *Leucochrysa (Nodita) azevedoi* was found to be relatively common in agricultural areas along Brazil’s Atlantic coast. The two other species are known only from their type localities: *Leucochrysa (Nodita) camposi* – coastal Ecuador, and *Leucochrysa (Nodita) morenoi* – Quito, Ecuador.

## Abstract (es)

Três species que Navás descreveu – *Leucochrysa (Nodita) azevedoi* Navás, 1913, *Leucochrysa (Nodita) camposi* (Navás, 1933) e *Leucochrysa (Nodita) morenoi* (Navás, 1934) – receberam atenção taxonômica recentemente. Essas espécies compartilham muitas características externas; de fato, o próprio Navás e outros autores posteriormente confundiram-se na sua identificação. Aqui, (a) identificações errôneas são corrigidas; (b) um neótipo de *Leucochrysa azevedoi* é designado; (c) *Leucochrysa (Nodita) morenoi*, sinonimizada previamente com *Leucochrysa (Nodita) camposi*, é reconhecida como espécie válida [**status reconduzido**], e (d) as três espécies são redescritas e ilustradas, com ênfase especial nos tipos. *Leucochrysa (Nodita) azevedoi* é relativamente comum em áreas agrícolas ao longo da costa atlântica do Brasil. As outras duas espécies são conhecidas apenas de sua localidade-tipo: *Leucochrysa (Nodita) camposi* – costa do Equador, e *Leucochrysa (Nodita) morenoi* – Quito, Equador.

## Introduction

Recent field surveys of natural enemies associated with insect pests in neotropical agroecosystems show that the chrysopid fauna is very rich and taxonomically complex (e.g., in Brazil: Freitas and Penny 2001, Multani 2008, Tauber et al. 2008). The Leucochrysines are especially diverse and difficult to identify; as a consequence, the literature on the group is rife with synonymies, errors, and uncertainty.

To help clarify the systematics of the group, we have begun examining the leucochrysine types and comparing them with recently collected or reared specimens. This is a slow process. Here we report the results of our studies with three *Leucochrysa* (*Nodita*) species that Navás described. All three resemble each other closely, and have been misidentified and confused with each other in both old and recent literature (Navás 1928, Freitas and Penny 2001, Legrand et al. 2008). One of these species occurs in agricultural settings throughout Brazil; thus timely clarification of its taxonomy is important.

## Taxonomy

### Leucochrysa (Nodita) azevedoi

Navás, 1913

http://species-id.net/wiki/Leucochrysa_(Nodita)_azevedoi

http://morphobank.org/permalink/?P243

**Figure 1.**
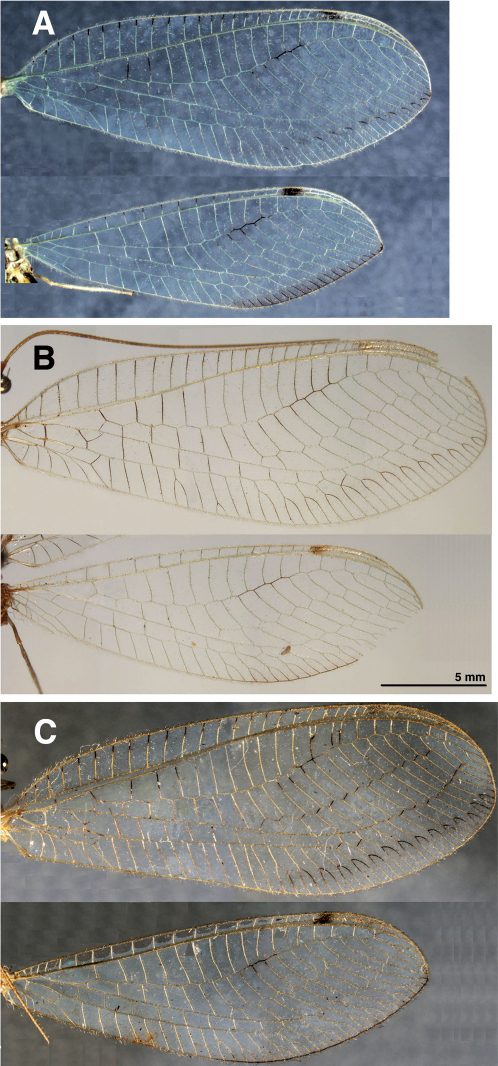
Wings. **A** *Leucochrysa (Nodita) azevedoi* (Male, Mato Grosso, Brazil, CAS) **B** *Leucochrysa (Nodita) camposi* (Male, Lectotype, MNHN) **C** *Leucochrysa (Nodita) morenoi* (Male, Lectotype, MNHN). Scale applies to all images.

Figs 1A 2 [Fig F3] [Fig F4] [Fig F5] 6

Leucochrysa azevedoi  Navás, 1913: 97; Type: missing; original description: “Brasil: Rio de Janeiro, Agosto de 1911. R.P. Joaquín da Silva Tavares S.J. (Col. m.)”. Navás (1912–1913: 303) [species list].
Nodita azevedoi  (Navás). First combination in *Nodita* by Navás (1928: 111) [collection record: Guayaquil, Ecuador, May 1926, probably misidentified; see below]. Navás (1929: 859) [collection record: Prov. de Rio de Jan., Coll. v. Bönninghausen, 20-X-1906, M. H., specimen probably destroyed]; Penny (1977: 25) [species list].
Leucochrysa (Nodita) azevedoi  Navás (Brooks and Barnard 1990: 277) [species list]; Oswald (2007) [catalog listing, nomenclature]; Legrand et al. (2008: 117) [probably Nomen Dubium]; Mantoanelli et al. (in press) [larval descriptions]. Here: confirmed as a valid species.

#### Type Material.

The original type remained in Navás collection (Navás 1913: 97); however, it does not exist in the Navás collection at the Natural History Museum of Barcelona now (Monserrat 1985: 240). Also, Legrand et al. (2008: 117) were unable to find it in the Muséum national d’Histoire naturelle, Paris (MNHN). It is reasonable to assume that the specimen has been destroyed; therefore, Legrand et al. (2008) considered the species a Nomen Dubium.

Recently we attempted to identify a *Leucochrysa* (*Nodita*) species from orchards in northern Rio de Janeiro State. Using the key by Freitas and Penny (2001), we identified the specimens as *Leucochrysa (Nodita) camposi*, a species that was described from coastal Ecuador; our specimens matched the drawings and description included with the key. However, comparison of our specimens with the type of *Nodita camposi* showed significant differences.

Earlier, we had noted that Navás had confused *Leucochrysa (Nodita) camposi* and *azevedoi* (see Legrand et al. 2008: 116). That is, he had labeled a specimen of *Leucochrysa (Nodita) camposi* in the MNHN as *Nodita azevedoi*; this specimen had been collected from the *Nodita camposi* type locality in 1930. [Note: Navás may also have similarly misidentified another specimen collected from the same locality in 1926 (Navás 1928: 111), but we have not seen that specimen.]

Navás’s errors then led us to consider whether our specimens could be *Leucochrysa (Nodita) azevedoi*. Indeed our specimens were collected in the state of Rio de Janeiro, where *Leucochrysa (Nodita) azevedoi* was originally collected; moreover our specimens fit Navás’s original description of *Leucochrysa azevedoi* well. Although Navás’s type of *Leucochrysa azevedoi* is missing, the similarities in external features, coupled with his confusion of the two species, give us confidence that Navás’s original description applies to the specimens we have at hand from northern Rio de Janeiro State.

As a result of our studies and the correction of Navás misidentifications, we now consider that *Leucochrysa (Nodita) azevedoi* ranges throughout coastal Brazil [and probably also into the state of Pará] and that *Leucochrysa (Nodita) camposi* occurs on the west coast of northern South America (currently known only from Ecuador). Now, to stabilize the nomenclature of these species, we designate as the *Leucochrysa azevedoi* neotype, a specimen (male) from the Brazilian state where the original type specimen was collected. Its labels read: (1) “Brazil: R[io de] J[aneiro], Campos dos Goytacazes, Est. Exp. PESAGRO (Estação Experimental da Empresa de Pesquisa Agropecuária do Estado do Rio de Janeiro) [21°44'55"S, 41°18'30"W], 06/IV/2002”; (2) “G. S. Albuquerque, Collector”; (3) “NEOTYPE *Leucochrysa azevedoi* Navás 1913, desig. C.A. Tauber, G.S. Albuquerque 2010.” In the Coleção Entomológica Pe. Jesus Santiago Moure (Universidade Federal do Paraná, Curitiba, PR, Brazil).

**Figure 2.**
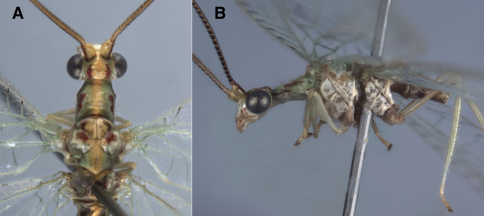
Habitus, *Leucochrysa (Nodita) azevedoi* (Male, Mato Grosso, Brazil, CAS). **A** Head, thorax, dorsal **B** Head, thorax, lateral.

**Figure 3. F3:**
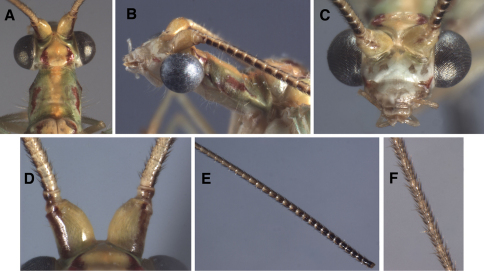
External features, *Leucochrysa (Nodita) azevedoi* (Male, Mato Grosso, Brazil, CAS). **A** Head, prothorax, dorsal **B** Head, prothorax, lateral **C** Head, frontal **D** Scapes, dorsal **E** Base of antenna, lateral **F** Mid-section of antenna.

#### Diagnosis.

**Figure 4. F4:**
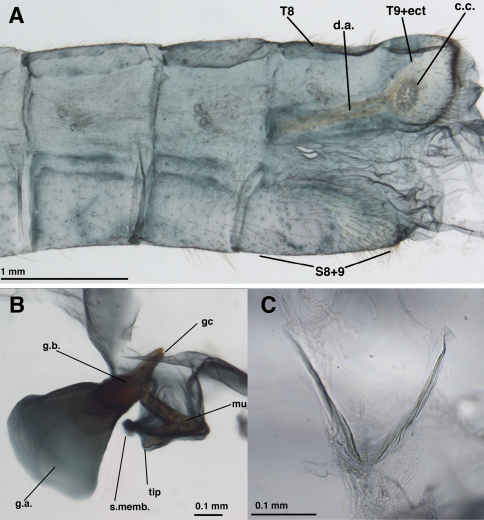
*Leucochrysa (Nodita) azevedoi*, Male (Rio de Janeiro, TRC). **A** Abdomen, lateral **B** Gonarcus, lateral **C** Hypandrium internum, dorsal. *Abbreviations:* c.c., callus cerci; **d.a.** dorsal apodeme **gc** gonocornu **g.a.** gonarcal apodeme **g.b** gonarcal bridge **mu** mediuncus **s.memb** setose membrane lateral to mediuncus beak **tip** tip of mediuncus beak **S8+9** fused eighth and ninth sternites **T8** eighth tergite **T9+ect** fused ninth tergite and ectoproct.

**Figure 5. F5:**
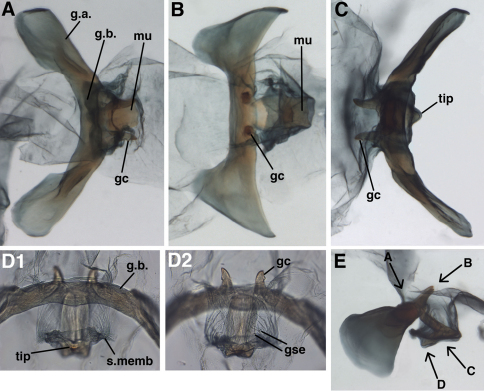
*Leucochrysa (Nodita) azevedoi*, Male gonarcus, series of views. The arrows on the lateral view, **E**, indicate the direction of the views in **A–D** (A-C, Rio de Janeiro, TRC; D, Mato Grosso, CAS). **A** View from above, with gonocornua projecting from the gonarcal bridge to the right, mediuncus extending downward, away from camera [Note heavy membrane above gonarcus extending over gonocornua] **B** Gonarcus with gonocornua projecting directly upward towards camera, mediuncus fully extended to right, covered with heavy membrane extending from gonarcal bridge **C** Gonarcus, view from beneath, with gonocornua extending to left **D** Mediuncus, view from below the tip of the beak: **D1** View at level of the beak; note the setose membranous areas lateral to the tip of the beak **D2** View at level above tip of beak; note membranous gonosaccus and gonosetae well above beak. *Abbreviations:* **gc** gonocornu **gse** gonoseta **g.a.** gonarcal apodeme **g.b.** gonarcal bridge **mu** mediuncus **s.memb**, setose membrane lateral to mediuncus beak **tip** tip of mediuncus beak.

**Figure 6.**
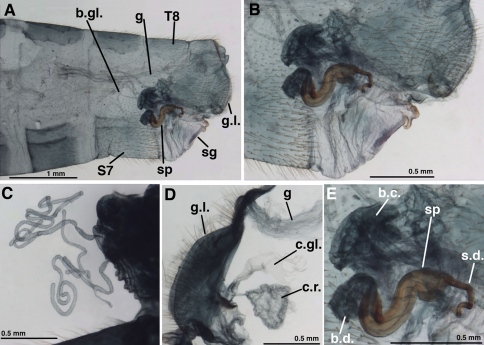
*Leucochrysa (Nodita) azevedoi*, Female (Rio de Janeiro, TRC). **A** Abdomen, lateral **B** Terminalia, lateral **C** Paired bursal glands [Note narrow connections to bursa copulatrix.] **D** Gonaphophyses laterales and colleterial structures [Colleterial gland damaged] **E** Spermatheca and part of bursa copulatrix. *Abbreviations:* **b.c.** bursa copulatrix **b.d.** bursal duct **b.gl.** bursal gland **c.gl.** colleterial gland **c.r.** colleterial reservoir **g** gut **g.l.** gonapophysis lateralis **sg** subgenitale **sp** spermatheca **s.d.** spermathecal duct **S7** seventh sternite **T8** eighth tergite.

Externally, *Leucochrysa (Nodita) azevedoi* adults closely resemble several *Leucochrysa* (*Nodita*) species that have a red to reddish brown dorsolateral stripe on the scape, dark brown to black ventrolateral marks on the basal flagellomeres, red to reddish brown marks on the sides of the raised portion of the vertex, a darkened section in the middle of the Radial sector (forewings and hindwings), and darkened terminal veinlets along the posteroapical margin of the hindwings (Figs 1A, 2–3). *Leucochrysa (Nodita) azevedoi* is slightly smaller than many of these species, including *Leucochrysa (Nodita) camposi* and *Leucochrysa (Nodita) morenoi* (wing length 17.7–19.5 mm versus ~21.3–21.6 for *Leucochrysa (Nodita) camposi* and *morenoi*), but it can only be identified with accuracy by its genitalic characters (male and female) (Figs 4–6). In the male *Leucochrysa (Nodita) azevedoi*, the gonarcal arms are broadly spread; they extend almost the full width of the segment. The gonocornua are located mesally on the gonarcal bridge, well within the span of the mediuncus, and they are directed upward from the bridge, not laterally from the bridge as are the *Leucochrysa (Nodita) camposi* gonocornua. The mediuncus is heavy; the beak is borne on a posteriorly projecting ventral plate (arcessus) that angles back towards the gonarcal arch; the beak is broad and blunt, and the membranous arms extending laterally from below the beak are rounded and each bears a lateral patch of setae. The hypandrium internum has a broad V-shape. Females can be recognized by their tubular spermatheca, small bursa, relatively small, fluted bursal duct. The bursa has two elongate, branched, tubular glands attached to its dorsal surface, and the subgenitale is heavy-based; the terminus has a thick neck, paired short, dorsal lobes and a short protrusion.

#### Adult description.

[Measurements: head, thorax, abdomen, wings (n=4), genitalia (n=2 mature males, 2 mature females)].

##### Head:

1.7–1.9 mm wide (including eyes); ratio of head width to eye width = 2.3–2.5:1. Vertex raised, with smooth surface, prominent, rounded posterior fold, without setae. Antenna 24.7 mm (~1.3 times length of forewing); scape longer than broad, (0.41–0.44 mm long, 0.32–0.34 mm wide), width = 3.2–4.4× distance between scapes, with three to four long setae distally on dorsal surface, shorter setae laterally; lateral margin fairly straight, mesal margin straight basally, curved outward distally; pedicel ~0.10 mm long, ~0.15 mm wide (at widest point); proximal flagellomeres short (segments 1, 2, 3: length = 0.85–1.0 times width), with three to four concentric rings of setae; middle and distal segments becoming longer (segments 6–8: length = 1.7–2.1 times width; distal segments: length = 3.0–3.2 times width), with four concentric rings of setae. Distance between scapes 0.08–0.10 mm; distance between tentorial pits 0.57–0.61 mm; length of frons (midway between scapes – midway between tentorial pits) 0.44–0.55 mm. Frons relatively flat mesally, with scalloped fold below toruli; surface smooth to slightly textured, with short setae. Clypeal margins straight; surface slightly textured, not horizontally striated. Labrum with distal margin indented mesally; dorsal surface smooth, rounded, setose distally. Ratio of genal length to distance between tentorial pits = 0.26–0.33:1.

##### Head coloration:

Antennae: scape cream to golden with light greenish tinge, broad red to reddish brown dorsolateral stripe extending full length of scape, onto anterolateral corner of dorsal torulus; pedicel cream with dark brown band distolaterally; flagellum cream with black setae, basal ~15 antennomeres with large dark brown to black mark ventrolaterally, fading on antennomeres ~16–20; marks forming prominent dark ventrolateral stripe. Vertex with central area raised, yellowish to greenish posteriorly, with prominent, triangular red to reddish brown mark laterally; area around raised area light green, unmarked. Frons, clypeus white, unmarked; labrum cream to amber; gena white, with narrow longitudinal red to reddish brown stripe immediately below tentorial pit. Torulus cream to amber. Maxilla, maxillary palpi, labium, labial palpi white to cream.

##### Thorax:

Cervix small, largely withdrawn below prothorax, light green, with small reddish brown lateral marks. Prothorax (sclerotized region) 0.86–0.93 mm long; 1.1–1.2 mm wide; ratio of length : width = ~0.69–0.77:1; setae thin, long, light golden; pronotum light green, with broad, golden yellow mesal stripe, with pair of lateral to sublateral elongate, red to reddish brown marks with irregular margins, sometimes with pair of small anterolateral reddish marks, pair of small, mesal reddish brown marks. Mesothorax, metathorax light green with golden yellow mesal stripe, sometimes with white; mesoprescutum with pair of crescent-shaped reddish brown marks; mesoscutum with pair of bold, submesal dark red to reddish brown marks; mesoscutellum unmarked; metascutum unmarked or with pair of small, diffuse, reddish brown spots mesally; metascutellum unmarked or with pair of small, diffuse reddish brown spots laterally. Legs unmarked, with golden setae; coxae, femora cream to white; tibiae light green, tarsi golden. Tarsal claws with deep cleft, elongate, with quadrate base.

##### Wings:

Forewing 17.7–19.5 mm long, 6.4–6.9 mm wide (at widest point); ratio of length : maximum width = 2.7–2.9:1. Costal area moderately broad; tallest costal cell (#7–9) 1.4–1.5 mm tall, 2.0–2.2 times width, 0.23–0.24 times width of wing (midwing). First intramedian cell triangular, 0.4–0.7 width of third median cell. First radial crossvein distal to origin of radial sector (Rs); radial area (between Radius and Rs) with single row of 16–18 closed cells; tallest cell (#7–8) 2.2–2.8 times taller than wide. No crassate veins; 5–6 b cells (= cells beneath Rs, not including an inner gradate vein). Two series of gradate veins; 9 inner gradates, 8–10 outer gradates; Eight b’ cells (cells beneath pseudomedia after second intramedian cell). Three intracubital cells (two closed). Membrane clear; stigma opaque, marked with brown. Veins green, with black on tips of costal veinlets, basal segment and midsection of Rs, two basal radial crossveins, distal arm of im2, bases of marginal forks.

Hindwing 15.3–16.9 mm long, 4.7–5.4 mm wide. Two series of gradate veins; 7–8 inner, 7–8 outer; 14–16 radial cells (counted from origin of Radius, not false origin). Five to six b cells (including small b1 cell); five to six b’ cells beyond second intramedian cell; three intracubital cells (two closed). Membrane clear, with posteroapical margin streaked with light brown; stigma pronounced, marked with brown basally. Veins mostly light green, but dark brown on tips of costal veinlets, midsection of Rs, tips of marginal forks, vein along distal half of posterior margin.

##### Abdomen:

Distal segments (beyond A4) moderately expanded; pleural region ca. twice height of sternites. Sternites, tergites with microsetae moderately dense; pleural region with very dense microsetae. Male: S6 approx. 1.2–1.4 times longer than tall, S7 approx. 1.0–1.1 times longer than tall (lateral view); female: S6 approx 1.4–1.7 times longer than tall, S7 approx. 1.7–2.0 times longer than tall. Tergites narrow, roughly rectangular, with lighter setae and longer microsetae than on sternites. Spiracles oval externally; atria not enlarged. Coloration: Dorsum with medial yellow stripe, deep red sublateral markings; sides green with deep red marks posterodorsally; venter white; callus cerci white; trichobothria pale.

##### Male:

Callus cerci oval to round, 0.20–0.27 mm diameter (range), with 29–37 relatively thin trichobothria of various lengths. Sternites 3–8 (not S1 or S2) with microtholi. T9+ectoproct truncate distally, fused mesally, midline without deep cleft, setae robust throughout, slightly smaller proximally than distally; ventral section of T9+ectoproct with broad, elongate proximal extension reaching full length of A8; proximal section well sclerotized, with apodeme heavy, unbranched, extending around proximal margin of callus cerci. S8+9 fused, without suture in mature specimens; S9 without microtholi; S8 much shorter, slightly taller than S9; S8+9 (lateral view) with proximal margin straight, rounded dorsally, acute ventrally; distal margin straight, with rounded apices, approximately 1/3 height of proximal margin. Setae on S9 slightly heavier than those on S5-S8; terminus of S9 without gonocristae. Subanal region membranous, with small striations around anus, no setae. Gonarcal complex connected to terminus of ectoproct by long, clear, smooth membrane that attaches to gonarcal bridge around base of gonocornua; gonocornua protruding through membrane; section of membrane below mediuncus extending interiorly and holding hypandrium internum. Gonarcus robust, very broadly arcuate, with lateral apodemes extending laterally almost entire width of T9+ect; gonarcal apodemes robust, stiff, slightly concave dorsally, rounded distally. Gonocornua broadly attached to gonarcal bridge, mesal to mediuncus, extending upward, away from mediuncus, short, stout, with inner margin curving outward, outer margin perpendicular to bridge, with lateral knob distally. Mediuncus largely membranous, dorsally with pair of longitudinal ridges, trough between, distally with pair of rounded lobes, recurved arcessus with heavy, rounded, beak-like tip; sides of mediuncus membranous, expanded, forming hollow depression; membrane inside depression bearing ~3 pairs of robust, medium-length setae on small chalazae; distal margins of mediuncal membrane with dense patches of relatively long, fine setae. Entoprocessus, tignum, gonapsis, pseudopenis, spinellae, gonocristae, gonosaccus absent. Hypandrium internum with broad V-shape, apex rounded, large, delicate, lightly sclerotized; comes elongate, thin, sometimes not visible.

##### Female:

Callus cerci round, 0.16–0.22 mm maximum diameter, with 25–33 trichobothria of mixed length. Tergite 8 roughly quadrate (lateral view), similar in depth to T6. Tergite 9+ectoproct elongate; posterior margin: dorsal half straight, almost perpendicular to dorsal margin of tergite, ventral half indented, angled inward, straight; ventral margin rounded, extending slightly below gonapophyses laterales. Sternite 7 with transverse weakness midlength, dorsal margin straight, not tapering distally; terminus unmodified, with terminal (posteroventral) setae slightly more dense, robust, and longer than other setae. Gonapophysis lateralis angled dorsally, rounded distally, ventrally, ~0.65–0.68 length of T9+ectoproct; inner membranous surface not expandable, with ~two vertical rows of short setae. Colleterial gland smooth-walled, delicate, with fluted, globate reservoir or secondary gland attached via a narrow duct immediately before transverse sclerification. Transverse sclerification robust, flat, platform-like with lateral margins upturned, with coarse longitudinal striations. Bursa copulatrix saccular, consisting of robust, densely folded membrane, extending anteriorly over spermatheca, into distal section of S7; pair of robust, branching, elongate, tubular bursal glands, connected to dorsolateral surface of bulbous end of bursa via short, very narrow duct. Spermatheca tubular, bent, thick at opening, tapering slightly (0.17–0.20 mm diameter at mouth, 0.12 mm diameter at midsection), 0.8–0.9 mm long, with broad, shallow (0.06–0.09 mm) invagination; slit along entire side of spermatheca opening to bursa. Spermathecal duct attached to anterior margin of spermatheca; basal section thick, straight, extending directly into base of subgenitale, then gradually tapering at first U-shaped bend, recurving posteriorly with two curves; basal ~one-half to two-thirds well sclerotized; distal section clear, brushy. Subgenitale broad, rounded, with smooth, rigid surface distally, with folded, membranous surface, with terminus broad, bilobed, with shallow, flat depression between lobes (heart shaped in posterior view); small ventral fold on S7 without setae.

#### Larvae.

Described elsewhere (Mantoanelli et al. in press).

#### Biology.

This species has been collected in orchards in the states of Rio de Janeiro, São Paulo, Mato Grosso and Rio Grande do Sul, in some cases in relatively large numbers. Its seasonal occurrence in orchards of coastal Brazil was assessed (Multani 2008).

#### Distribution.

Currently known only from Brazil. We have seen specimens from the States of Rio de Janeiro, Matto Grosso, and São Paulo [also see Freitas and Penny 2001, as *Leucochrysa (Nodita) camposi*]. Probably, the species also occurs in the State of Pará. [Note: Adams (1987) considered a specimen that he studied from Pará (illustrated in Fig. 18) to be an abnormal variant of *Leucochrysa (Nodita) amazonica* (Navás). It is not clear why he did so; Navás’s description of *Leucochrysa (Nodita) amazonica* was based on a single teneral male from the lower Amazon. Our study shows that Adams’ figure of the specimen from Pará is typical of *Leucochrysa (Nodita) azevedoi*.]

#### Variation.

See images on our project site at www.morphobank.org. In teneral males, S8 and S9 are only partially fused; the segments are fully fused, but well demarcated in mature males. When teneral, the gonarcus is thin and delicate; the gonocornua are small and closely aligned, adjacent to the mediuncus; and the setose lateral extensions below the beak of the mediuncus are not visible, but the beak is distinct.

In addition to the specimens listed below, we examined one specimen (a male) from the State of Rio Grande do Sul. This specimen’s background color and markings are lighter than those of the other specimens; in addition, the beak on its mediuncus is sharp and straight, not blunt and decurved as in *Leucochrysa (Nodita) azevedoi*. A determination of whether this specimen represents a separate species or a variant population of *Leucochrysa (Nodita) azevedoi* awaits the availability of additional specimens.

#### Adult specimens examined.

In addition to the neotype listed above: Brazil. Rio de Janeiro: Campos dos Goytacazes, VI/22/1999, R. R. Moraes (1♂, 1♀, Tauber Research Collection, TRC); Campos dos Goytacazes, Est. Exp. PESAGRO (Estação Experimental da Empresa de Pesquisa Agropecuária do Estado do Rio de Janeiro, 21°44'55"S, 41°18'30"W, 14m, VII-2005 – III-2007, G. S. Albuquerque, J. S. Multani, Collectors (2♂♂, 2♀♀, alcohol, UENF; 3♂♂, 3♀♀, alcohol, 1♀ pinned, TRC). São Paulo: Jaboticabal, FCAV, 10 July 1998, Freitas, S., 10 (1♂, California Academy of Sciences, CAS). Mato Grosso: Itiquira, P. E. Michelin, 18 July 1998, Freitas, S., M10 (1♂, CAS).

#### Etymology.

Latinized proper name, genitive. Navás named the species in honor of Ignacio de Azevedo (1528–1570), a Portuguese Jesuit who labored in Brazil and was killed along with 39 Jesuit companions, while sailing near the Canary Islands on a return voyage from Rome to Brazil. They are known as the “Fourty Martyrs of Brazil”.

### Leucochrysa (Nodita) camposi

(Navás, 1933)

http://species-id.net/wiki/Leucochrysa_(Nodita)_camposi

http://morphobank.org/permalink/?P243

Figs 1B 7 [Fig F8] [Fig F9] 10

Nodita camposi  Navás, 1933: 197–8, Fig. 17 (Lectotype: MNHN, male, examined; original description: “Ecuador: Guayaquil, Julio de 1932. Campos R. Leg.”). Navás (1934b: 16) [collection record: “Guayaquil (Ecuador), 1933. Campos leg.”; specimen in MNHN]; Penny (1977: 25) [species list].
Leucochrysa (Nodita) camposi  (Navás). First combination in *Leucochrysa* (*Nodita*) apparently by Brooks and Barnard (1990: 277) [species list]. Freitas and Penny (2001: 287) [misidentified; see *Leucochrysa (Nodita) azevedoi*, above]; Oswald (2007) [catalog listing, nomenclature]; Legrand et al. (2008: 121) [lectotype designation, information on type].
Nodita morenoi Navás 1934a: 157–158 (Lectotype: MNHN, male, examined; original description: “Équateur, Quito, R. Benoist, 1930. Mus. París.”). Synonymized by Legrand et al. (2008: 154). Here: **Synonymy reversed** (see below).

#### Type material.

The *Nodita camposi* lectotype, a male, is badly discolored, but its wings are in good condition, its body is mature, and the genitalia are well sclerotized. A female specimen collected one year later, at the same locality as the type, is also mature.

#### Diagnosis.

*Leucochrysa (Nodita) camposi* can be distinguished from *Leucochrysa (Nodita) azevedoi* by its somewhat larger size and more robust appearance (see above) and genitalic characters (male and female) (Figs 8–10). In the male *Leucochrysa (Nodita) camposi*, the gonarcal arms are narrowly arched; the gonarcal arms extend perpendicularly from the gonarcal bridge, not laterally as in *Leucochrysa (Nodita) azevedoi*. The gonocornua are located distally on the gonarcal bridge, well outside the span of the mediuncus, and they extend laterally away from the gonarcal bridge. Like *Leucochrysa (Nodita) azevedoi*, the mediuncus is heavy apically and its beak is borne on a plate (arcessus) that bends back toward the gonarcal arch; however, the membrane extending from the beak does not have distinct arms or patches of setae, as are found on *Leucochrysa (Nodita) azevedoi*. The hypandrium internum is delicate and has a very narrow U-shape. The *Leucochrysa (Nodita) camposi* female can be recognized by its bulbous spermatheca, asymmetrical bursa that extends from its left side over the spermatheca and part of the bursal duct, and its highly coiled bursal duct that extends well into the sixth abdominal segment. The bursal glands are elongate, branching and ribbon-like.

#### Adult description.

##### Head:

1.9 mm wide (including eyes); ratio of head width to eye width = 2.1:1. Vertex raised, with smooth surface, four prominent, circular muscle attachment scars along posterior margin, without setae. Antenna 33.3 mm (~1.5 times length of forewing); scape longer than broad, (0.47–0.50 mm long, 0.36–0.39 mm wide), width = 4.2–4.5× distance between scapes, with ~four long setae distally on dorsal surface, shorter setae laterally; lateral margin fairly straight, mesal margin straight basally, curved outward distally; pedicel ~0.17 mm long, ~0.17 mm wide (at widest point); proximal flagellomeres short (segments 1, 2, 3: length = 1.2–1.5 times width), with three concentric rings of setae; middle and distal segments becoming longer (segments 8–10: length = 1.5–1.8 times width; distal segments: length = 2.1 times width), with four concentric rings of setae. Distance between scapes 0.09 mm; distance between tentorial pits 0.65 mm; length of frons (midway between scapes – midway between tentorial pits) 0.50 mm. Frons relatively flat mesally, with scalloped fold below toruli; surface smooth. Clypeal margins straight; surface slightly textured, not horizontally striated. Labrum with distal margin slightly indented mesally; dorsal surface smooth, setose distally; sides rounded. Ratio of genal length to distance between tentorial pits = 0.31:1.

##### Head coloration.

Specimen largely discolored with age. Antennae: scape amber colored mesally, noticeably darker laterally, probably a broad reddish brown dorsolateral stripe extending full length of scape; pedicel probably with brownish band distolaterally; flagellum cream with golden brown setae, basal ~20 antennomeres with large dark brown to black mark ventrally, fading on antennomeres ~20–28; marks forming prominent dark stripe ventrally. Vertex with raised central area discolored, with thin reddish brown streak across frontal margin, trace of large triangular red mark laterally; area between raised area and eyes maybe marked with red. Frons, clypeus white, unmarked; labrum cream to amber; gena discolored, possibly red throughout. Maxilla, maxillary palpi, labium, labial palpi white to cream.

##### Thorax:

Cervix small, largely withdrawn below prothorax, discolored, with small reddish lateral marks. Prothorax (sclerotized region) 0.95 mm long; 1.3 mm wide; ratio of length : width = 0.74:1; prothorax (extended) 1.3 mm long; setae thin, long, golden; discolored, with pair of anterolateral spots, pair of elongate, sublateral, red marks with irregular margins. Mesothorax, metathorax discolored; mesoprescutum with pair of red marks mesally; mesoscutum with pair of red marks on anteromesal flank; metascutum with pair of thin red submesal streaks from middle to posterior margin. Legs unmarked, with golden setae, described by Navás as: greenish, tarsi probably amber.

##### Wings:

Forewing 21.3–21.6 mm long, 8.1–8.2 mm wide (at widest point); ratio of length : maximum width = 2.7:1. Costal area slightly expanded basally; tallest costal cell (#8, 9) 1.1 mm tall, 2.1 times width, 0.2 times width of wing (midwing). First intramedian cell triangular, 0.7 times width of third median cell. First radial crossvein distal to origin of radial sector (Rs); radial area (between Radius and Rs) with single row of 17 closed cells; tallest cell (#6) 3.0 times taller than wide. No crassate veins; 5 b cells. Two series of gradate veins; 10–11 inner gradates, 10–11 outer gradates; 6–8 b’ cells. Three intracubital cells (two closed). Membrane clear, except posteroapical faint tinge of brown streaked across bases of forked distal veinlets; stigma golden, with small brown mark basally. Veins green, with dark brown to black on tips of costal veinlets, basal segment of Rs (to first posterior crossvein), two basal radial crossveins, midsection of Rs and radial crossveins above, bases of marginal forks.

Hindwing 18.5–18.8 mm long, 5.5–6.1 mm wide. Two series of gradate veins; 8 inner, 9 outer; 15 radial cells (counted from origin of Radius, not false origin). Four to five b cells (including small b1 cell); five b’ cells beyond second intramedian cell; two intracubital cells (one closed). Stigma golden, marked with brown distally; membrane clear with posteroapical margin streaked with faintly brown tinge. Veins green, except midsection of Rs, tips of marginal forks (posteroapical margin of wing) dark brown to black.

##### Abdomen

(male type only; female abdomen damaged): Distal segments (beyond A4) expanded; pleural region (P6) ca. 2.75 times height of sternite (S6). Setae on tergites, sternites, moderately long, slender; setae on pleura short, small; microsetae moderately dense, small throughout. S6 approx. 1.3 times longer than tall, S7 approx. 1.1 times longer than tall (lateral view). Tergites cap-like, with lighter setae and longer microsetae than on sternites. Spiracles oval externally; atria not enlarged. Coloration: specimen discolored.

##### Male

[gonarcal complex separated from abdomen, connecting membrane remains attached to gonarcus]: Callus cerci ~round, 0.21–0.25 mm diameter (range), with 39 long, thin, trichobothria. Sternites 3–8 (not S1 or S2) with microtholi. T9+ectoproct short, truncate distally, fused mesally, midline without deep cleft, setae long, moderately slender throughout; ventral section of T9+ectoproct with elongate proximal extension reaching full length of A8; proximal section well sclerotized, with apodeme heavy, extending around proximal margin of callus cerci. S8+9 fused, without suture; S9 without microtholi; S8 much shorter, slightly taller than S9; S8+9 (lateral view) with proximal, distal margins straight, with acute ventral apices, rounded dorsal apices, slightly <1/2 height of proximal margin. Setae on S9 very slightly heavier than those on S5-S8; terminus of S9 without gonocristae. Subanal region membranous, with small striations around anus, no setae. Gonarcal complex attached to gonarcal bridge by clear, smooth membrane around base of gonocornua; gonocornua protruding through membrane; section of membrane below mediuncus confluent with gonosaccus below mediuncus, holding hypandrium internum distally. Gonarcus robust, narrowly arcuate, with lateral apodemes extending perpendicularly from gonarcal bridge; gonarcal apodemes robust, stiff, lateral surface slightly concave, expanded distally (lateral view). Gonocornua broadly attached to gonarcal bridge distal to mediuncus, broad basally, tapering distally, with straight margins, rounded tips. Mediuncus largely sclerotized; base with membranous attachment to dorsum of gonarcal bridge, mesal to gonocornua; basal section with pair of shallow, elongate depressions, acute ridge between, becoming flattened, expanded distally; terminus rounded laterally, scalloped frontally; ventral surface (below expanded tip of mediuncus) with elongate, recurved, mesal beak; sides of mediuncus membranous, connecting below mediuncus, forming internal pouch below beak, above gonosaccus. Gonosaccus clear, smooth, folded membrane, bearing ~3 pairs of robust, medium-length gonosetae on small chalazae. Entoprocessus, tignum, gonapsis, pseudopenis, spinellae, gonocristae absent. Hypandrium internum with narrow U-shape, apex rounded, delicate, lightly sclerotized; comes very faint.

##### Female:

Callus cerci round, 0.26 mm maximum diameter, with 39 slender trichobothria. Tergite 8 roughly quadrate (lateral view), similar in depth to T6. Tergite 9+ectoproct elongate; posterior margin: dorsal one-third straight, perpendicular to dorsal margin of tergite, then indented, angled inward, straight; ventral margin rounded, extending to depth of gonapophyses laterales. Sternite 7 largely damaged. Gonapophysis lateralis angled dorsally, rounded distally, ventrally, ~0.60 length of T9+ect; inner membranous surface not expandable, with sparse, small setae on inner surface. Colleterial gland missing but short, thick duct attached immediately before transverse sclerification. Bursa copulatrix concave, shield-like, lightly folded, thick membranous sac, extending anteriorly over spermatheca, into mid-section of S7, also extending, asymetrically on left side of S7 above ca. basal one-half of coiled bursal duct; with pair of slender, elongate, branching, ribbon-like bursal glands, one connected on either side of bursa, near interior tip of bursa. Bursal duct flat to thickened, coiled, robust, tapering anteriorly to A6, then coiling back on itself to bursa; membranous throughout. Spermatheca broad, thick, U-shaped tube, thick walled with wide circular opening facing subgenitale, enlarged tire-shaped bulge at open end, prominent slit along entire dorsum, opening to bursal duct (0.16 mm diameter at mouth, narrower at midsection), ~0.5 mm long, with narrow (0.04 mm), moderate length (0.12 mm) invagination. Spermathecal duct uncolored, originating on dorsal surface of spermatheca at terminus of dorsal slit, making two sharp bends, a U-shaped bend in subgenitale, then glandulose thicked portion making broad bend back toward spermatheca. Subgenitale large, rounded, with rigid, folded, heavy membraneous texture, terminus broad, flat-surfaced, bilobed dorsally (heart shaped in posterior view), with large, flat transverse depression mesally below lobes, with ventrally protruding tip; small ventral fold above S7 without setae.

**Figure 7.**
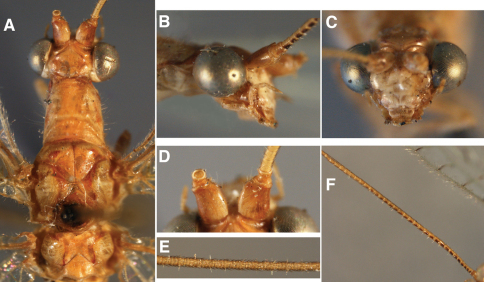
External features, *Leucochrysa (Nodita) camposi*, Male (Guayaquil, Ecuador, Lectotype, MNHN). **A** Head, thorax, dorsal **B** Head, lateral **C** Head, frontal **D** Scapes, dorsal **E** Mid-section of antenna **F** Base of antenna, dorsal. Note red markings on the notum of each segment.

**Figure 8. F8:**
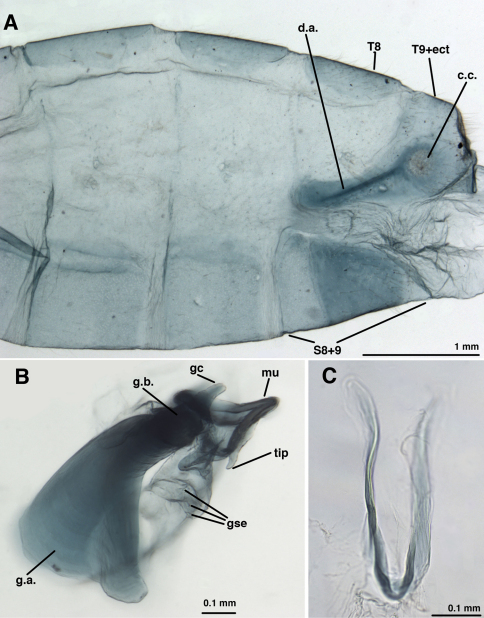
*Leucochrysa (Nodita) camposi*, Male (Guayaquil, Ecuador, Lectotype, MNHN). **A** Abdomen, lateral **B** Gonarcus, lateral **C** Hypandrium internum, dorsal. *Abbreviations:* **c.c.** callus cerci **d.a.** dorsal apodeme **gc** gonocornu **gse** gonoseta **g.a.** gonarcal apodeme **g.b.** gonarcal bridge **mu** mediuncus **tip** tip of mediuncus beak **S8+9** fused eighth and ninth sternites **T8** eighth tergite **T9+ect** fused ninth tergite and ectoproct.

**Figure 9. F9:**
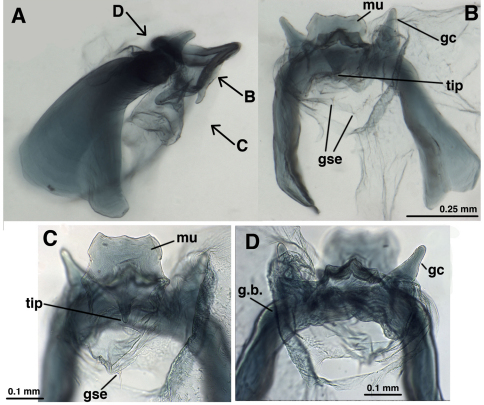
*Leucochrysa (Nodita) camposi*, Male gonarcus, series of views. The arrows on the lateral view, **A**, indicate the direction of the views in **B–D**. (Guayaquil, Ecuador, Lectotype, MNHN). **A** Lateral view **B** Gonarcus, view from beneath, with gonocornua extending upward above gonarcal bridge [Note heavy membrane around base of gonocornua] **C** Gonarcus, view from beneath, with gonocornua extending upward above gonarcal bridge, focus on frontal surface of mediuncus and gonoseta on membrane beneath gonarcus **D** Gonarcus, view from above, with gonocornua extending upward above gonarcal bridge, focus on junction of mediuncus with gonarcal bridge. *Abbreviations:* **gc** gonocornu **gse** gonoseta **g.b.** gonarcal bridge **mu** mediuncus **tip** tip of mediuncus beak.

**Figure 10.**
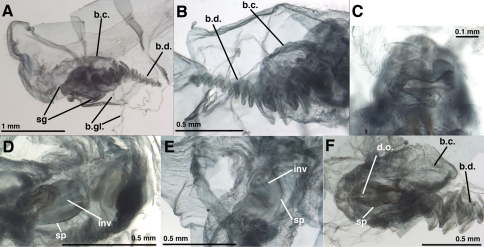
*Leucochrysa (Nodita) camposi*, Female [MNHN, Nontype Navás specimen]. **A** Terminalia, dissected, lateral view from right **B** Bursal duct, part of bursa copulatrix, lateral view from left **C** Subgenitale, posterior view **D** Spermatheca beneath bursa copulatrix, ventral view **E** Spermatheca beneath bursa copulatrix, ventrolateral view **F** Spermatheca, dorsal view, through bursal copulatrix [Note dorsal slit.]. *Abbreviations:* **b.c.** bursa copulatrix **b.d.** bursal duct **b.gl.** bursal gland **d.o.** dorsal slit or opening from spermatheca to bursa copulatrix **inv** spermathecal invagination **sg** subgenitale **sp** spermatheca.

#### Larvae and biology.

Unknown.

#### Distribution.

Currently known only from Ecuador (Guayaquil, Quito).

#### Adult specimens examined.

In addition to the type listed above, we examined two female specimens from the type locality; both are in the MNHN. One was collected in 1933 and was reported by Navás (1934b: 16). The other specimen bears a determination label reading “Nodita Azevedoi Nav, P. Navás S.J. det.”; it was reported by Navás (1928: 111). The descriptions above are based on the male type and the female specimen reported by Navás in 1934.

#### Etymology.

Navás named the species in honor of its collector, D. Francisco Campos R. of Guayaquil, Ecuador. Campos provided Navás with numerous lacewing specimens from Ecuador.

### Leucochrysa (Nodita) morenoi

(Navás, 1934)

http://species-id.net/wiki/Leucochrysa_(Nodita)_morenoi

http://morphobank.org/permalink/?P243

Figs 1C 11 [Fig F12] 13

Nodita morenoi  Navás, 1934a: 157–158 (Lectotype: MNHN, male, examined; original description: “Équateur, Quito, R. Benoist, 1930. Mus. París.”); Penny (1977: 26) [species list].
Leucochrysa morenoi  (Navás) – listed by Oswald (2007). We could not find a supporting reference.
Leucochrysa (Nodita) morenoi  (Navás). First combination in *Leucochrysa* (*Nodita*) apparently by Brooks and Barnard (1990: 277) [species list]. Oswald (2007) [catalog listing, nomenclature]; Legrand et al. (2008: 154) [lectotype designation, information on type, identified as subjective junior synonym of *Leucochrysa (Nodita) camposi* (Navás, 1933)]. Here: **Reinstated as a valid species.**

#### Type material.

The *Nodita morenoi* lectotype, a male, is discolored, but otherwise in good condition; the abdomen is cleared and in a vial with glycerin. For this study, we re-examined the types of *Nodita morenoi* and *Nodita camposi* side-by-side; here, we provide images and a re-description to support our recognition of *Leucochrysa (Nodita) moreni* as a valid species.

#### Diagnosis.

**Figure 11.**
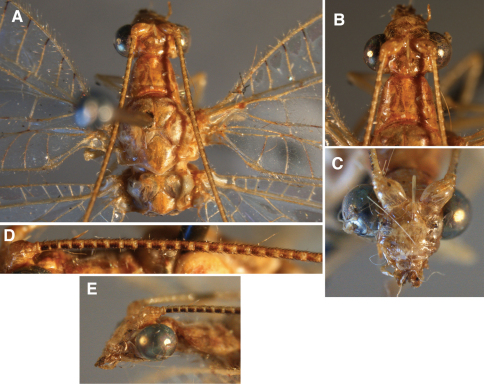
External features, *Leucochrysa (Nodita) morenoi*, Male (Quito, Lectotype, MNHN). A. Head, thorax, dorsal; B. Head, prothorax, dorsal; C. Head, frontal; D. Antennal base, lateral; E. Head, lateral.

**Figure 12. F12:**
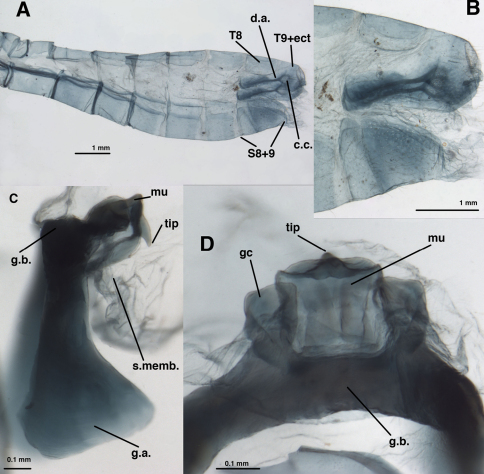
*Leucochrysa (Nodita) morenoi*, Male (Quito, Lectotype, MNHN). **A** Abdomen, lateral **B** Terminalia, lateral **C** Gonarcus, lateral **D** Gonarcus, dorsal view. *Abbreviations:* **c.c.** callus cerci **d.a.** dorsal apodeme **gc** gonocornu **g.a.** gonarcal apodeme **g.b.** gonarcal bridge **mu** mediuncus **s.memb** setose membrane lateral to mediuncus beak **tip** tip of mediuncus beak **S8+9** fused eighth and ninth sternites **T8** eighth tergite **T9+ect** fused ninth tergite and ectoproct.

**Figure 13.**
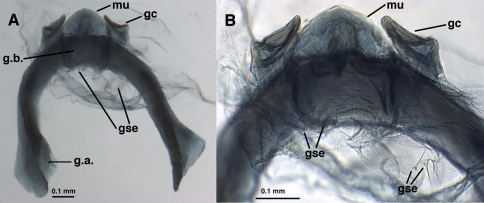
*Leucochrysa (Nodita) morenoi*, Male (Quito, Lectotype, MNHN). **A, B** Gonarcus, ventral view. *Abbreviations:* **gc** gonocornu **gse** gonoseta **g.a.** gonarcal apodeme **g.b.** gonarcal bridge **mu** mediuncus.

*Leucochrysa (Nodita) morenoi* is known only from the type specimen, which is badly discolored with age. At this time, the only reliable way to identify specimens is with the male genital characters (Figs 12 & 13). In both species the gonarcal arch is U-shaped, with the gonarcal apodemes approximately perpendicular to the bridge. However, unlike in *Leucochrysa (Nodita) camposi*, the *Leucochrysa (Nodita) morenoi* gonarcus is rounded posteriorly (c.f. Fig. 12C with Fig. 8B); the mediuncus is rounded dorsally; and the gonocornua are flattened and expanded laterally (c.f. Figs 12 and 13 with Fig. 9). Externally (Fig. 11), *Leucochrysa (Nodita) morenoi* resembles a large number of species, including *Leucochrysa (Nodita) azevedoi* and *Leucochrysa (Nodita) camposi*, in the following traits: dark brown to black, lateral marks on the basal ~15 flagellomeres that give impression of a streak on the exterior edge of the antenna, a darkened section in the middle of the Radial sector (forewings and hindwings), and darkened terminal veinlets along the posteroapical margin of the hindwings. The head and thorax are too discolored to determine if there are reddish markings on the vertex, gena, scape, or prothorax as there are on many of the other species with these traits. We suspect that they are not present on this species because the only marks that Navás (1934a) mentioned in the original description were an iron-grey color on the lateral margins of the pronotum (not visible on the type now) and a pair of dark brown (“fuscis”) marks on the mesoprescutum (present on the type). *Leucochrysa (Nodita) morenoi* is large and robust in appearance, more like *Leucochrysa (Nodita) camposi*, than *Leucochrysa (Nodita) azevedoi* [e.g., forewing length: 21–22 mm for *Leucochrysa (Nodita) morenoi* and *Leucochrysa (Nodita) camposi* versus 18–19.5 mm for *Leucochrysa (Nodita) azevedoi*].

#### Adult description.

##### Head:

2.12 mm wide (including eyes); ratio of head width to eye width = 1.85:1. Vertex approximately oval, raised slightly, with smooth to slightly textured surface, small posterior fold. Antenna 38 mm long (~1.8 times length of forewing); scape longer than broad, (~0.4 mm wide), width = 2× distance between scapes, with long setae distally on dorsal surface, shorter setae laterally; lateral margin fairly straight, mesal margin straight basally, curved outward distally; pedicel ~0.25 mm long, ~0.18 mm wide (at widest point); proximal flagellomeres short (segments 1, 2, 3: length = 1.2–1.5 times width), with three to four concentric rings of setae; middle and distal segments becoming longer (segments 9–11: length = 1.6–1.7 times width; distal segments: length = 2.2–2.6 times width), with four concentric rings of setae. Distance between scapes 1.04 mm; distance between tentorial pits 0.65 mm; length of frons (midway between scapes – midway between tentorial pits) 0.77 mm. Frons relatively flat, with slightly scalloped fold below toruli; surface smooth to slightly textured. Clypeus straight distally; surface slightly textured, not horizontally striated. Labrum with distal margin very slightly concave mesally; dorsal surface smooth, rounded, setose distally. Ratio of genal length to distance between tentorial pits = 0.41:1.

##### Head coloration:

Antennae pale, probably yellow or cream, with or without lateral stripe on scape; pedicel pale, possibly with darkened ring distally; flagellum pale with dark brown to black setae; basal ~15 antennomeres with dark brown lateral marks, fading on antennomeres 16-~20. Vertex and remainder of head pale, with no discernable markings. Frons, clypeus white, unmarked; gena discolored, probably without markings. Torulus cream. Maxilla, maxillary palps, labium, labial palps pale, unmarked.

##### Thorax:

Cervix small, largely withdrawn below prothorax, discolored. Prothorax (sclerotized region) 0.83 mm long; 1.03 mm wide; ratio of length : width = 0.56:1; prothorax (extended) 1.5 mm long; setae thin, long, golden; surface discolored, scattered patches of red tinge visible, but not mentioned in original description, small grey area mentioned in original description not apparent now. Mesothorax, metathorax discolored; mesoprescutum with pair of sublateral reddish brown marks (mentioned in original description); mesoscutum with pair of brown streaks on anterior and posterion margin; mesoscutelllum unmarked; metascutum with pair of brownish spots on posterior margin. Legs unmarked, with golden setae, originally described as greenish.

##### Wings:

Forewing 21.6 mm long, 8.0 mm wide (at widest point); ratio of length : maximum width = 2.7:1. Costal area moderately broad; tallest costal cell (#10) 1.4 mm tall, 1.8 times width, 0.19 times width of wing (midwing). First intramedian cell triangular, 0.6 width of third median cell. First radial crossvein distal to origin of radial sector (Rs); radial area (between Radius and Rs) with single row of 17 closed cells; tallest cell (#5) 2.8 times taller than wide. No crassate veins; 6 b cells. Two series of gradate veins; 13 inner gradates, 11 outer gradates; nine b’ cells. Three intracubital cells (two closed). Membrane clear; stigma golden, marked with brown basally. Veins mostly green (probably), with dark brown on tips of most costal veinlets, basal segment, three basal radial crossveins, small midsection of Rs, crossveins stemming from darkened midsection, inner gradates, outer gradates, bases of marginal forks.

Hindwing 18.7 mm long, 6.2 mm wide. Two series of gradate veins; 9 inner, 9 outer; 17 radial cells (counted from origin of Radius, not false origin). Six b cells (including small b1 cell); seven b’ cells beyond second intramedian cell; two intracubital cells (two closed). Membrane clear, probably streaked with light brown tinge along posterodistal margin; stigma pronounced, marked with brown. Veins mostly green (probably), but dark brown on midsection of Rs, tips of crossveins extending from darkened midsection, and posteroapical margin of wing.

##### Abdomen:

Distal segments (beyond A4) expanded; pleural region ca. two times height of sternites. Sternites, tergites with microsetae relatively sparse; male: S6 ca. same height as length, S7 approx. 1.1 times taller than long (lateral view). Tergites roughly rectangular, with rounded posterior and lateral margins, shorter setae than on sternites. Spiracles oval externally; atria not enlarged. Coloration: information not available.

##### Male:

Callus cerci ca. round, 0.25–0.27 mm diameter (range), with 35, 36 relatively long, thin trichobothria. Sternites 5–8 with microtholi; S1–4 without. Dorsum of T9+ectoproct truncate distally, fused mesally, midline without deep cleft, setae short, delicate throughout; ventral section of T9+ectoproct with elongate proximal extension reaching full length of A8; proximal section broad, well sclerotized, with apodeme slender, slightly bent in center, extending around posterior margin of callus cerci; dorsal arm of apodeme extending above callus cerci, with acute tip; ventral arm of apodeme extending around callus cerci becoming diffuse posteriorly. S8+9 fused, with distinct demarcation, without suture; S9 without microtholi; S8 shorter than S9, ca. same height as base of S9; S8+9 (lateral view) with proximal margin straight, rounded dorsally, acute ventrally; distal margin straight, with rounded apices, approximately 0.7 times height of proximal margin. Setae on S9 mostly broken, probably slightly heavier than those on S5-S8; terminus of S9 without gonocristae. Subanal region membranous, with flat membrane below anus, no setae. Gonarcal complex removed from abdomen, membranes not intact; gonarcal membrane extending from top of gonarcus, converging with gonosaccus membrane. Gonarcus, from dorsal, ventral views: U-shaped, robust, with apodemes extending perpendicularly, smoothly, from ends of rounded gonarcal bridge; gonarcal bridge, in lateral view: broad, with straight, parallel margins, distal one-half expanding into apodemes that extend below mediuncus. Gonocornu short, flat in lateral view, broad, squareish in posterior view, extending from top of gonarcal bridge, scalloped around mediuncus. Mediuncus with dome-like base, extending foreward from below and mesal to gonocornua; terminus truncate, with large, beak-like tip between pair of rounded distal lobes; top of mediuncus with two broad, scalloped, rod-like sclerotized depressions extending entire length from base at gonarcal bridge to base of beak; ventral surface rugose, hollow, covered by thin, tight membrane that merges with gonosaccus. Gonosaccus extending from below gonarcal bridge, with three pairs of lateral gonosetae. Area below gonarcal complex, hypandrium internum missing. Entoprocessus, tignum, gonapsis, pseudopenis, spinellae and gonocristae absent.

##### Female:

Unknown.

#### Larvae and biology.

Unknown.

#### Distribution.

Currently known only from Quito, Ecuador.

#### Adult specimens examined.

Known only from the type.

#### Etymology.

Navás named this species in honor of President Gabriel García Moreno of Ecuador (1821–1875). Moreno was a noted statesman, who strongly supported education, science, and the Roman Catholic Church.

## Keys to species

The latest key to South American *Leucochrysa* species relies on external features of adults to identify species that occur in Brazilian agroecosystems (Freitas and Penny 2001: 285). As explained above, this key identifies specimens of *Leucochrysa (Nodita) azevedoi* as *Leucochrysa (Nodita) camposi*. Thus, the name *Leucochrysa (Nodita) azevedoi* should replace *Leucochrysa (Nodita) camposi* in cuplet 9 of the key. *Leucochrysa (Nodita) camposi* is not known from Brazil and therefore should not be included in Freitas and Penny’s treatment.

Unfortunately, each of the other two species — *Leucochrysa (Nodita) camposi* and *Leucochrysa (Nodita) morenoi* – are known from only a single specimen each (the types), and these specimens are too badly discolored to be useful in incorporating the species into a key using external characters. Reliable identifications require dissection of male and/or female genitalia, and at this time the genitalia of too few species have been studied to develop a useful key.

All three species treated here appear to be related to *Leucochrysa (Nodita) amazonica* and its relatives (see Adams 1987, Freitas and Penny 2001); they are large bodied, apparently have similar body markings, and share a truncated plate at the tip of the mediuncus (arcessus of Freitas and Penny 2001) and elongate, coiled bursal ducts. Future studies should include comparisons among the species that share these traits.

## Supplementary Material

XML Treatment for Leucochrysa (Nodita) azevedoi

XML Treatment for Leucochrysa (Nodita) camposi

XML Treatment for Leucochrysa (Nodita) morenoi
